# Registered psychiatric service use, self-harm and suicides of children and young people aged 0–24 before and during the COVID-19 pandemic: a systematic review

**DOI:** 10.1186/s13034-022-00452-3

**Published:** 2022-02-25

**Authors:** Wan Mohd Azam Wan Mohd Yunus, Laura Kauhanen, Andre Sourander, June S. L. Brown, Kirsi Peltonen, Kaisa Mishina, Lotta Lempinen, Kalpana Bastola, Sonja Gilbert, David Gyllenberg

**Affiliations:** 1 Research Centre for Child Psychiatry, Department of Child Psychiatry, University of Turku, Lemminkäinenkatu 3, 3rd. floor, 20014 Turku, Finland; 2 INVEST Research Flagship, University of Turku, Turku, Finland; 3 Department of Psychology, Faculty of Social Sciences and Humanities, Universiti Teknologi Malaysia, Johor Bahru, Johor Malaysia; 4 Department of Child Psychiatry, Turku University Hospital, Turku, Finland; 5 Department of Psychology, Institute of Psychiatry, Psychology & Neuroscience (IoPPN), King’s College London, London, England, UK; 6 Unit of Health Sciences, Faculty of Social Sciences, Tampere University, Tampere, Finland; 7 National Institute for Health and Welfare, Helsinki, Finland; 8 Department of Adolescent Psychiatry, Helsinki University Central Hospital, Helsinki, Finland

**Keywords:** Service use, COVID-19 pandemic, Children, Adolescents, Young people, Mental health, Psychiatric, Suicide

## Abstract

**Background:**

The COVID-19 pandemic has impacted on psychiatric symptoms of children and young people, but many psychiatric services have been disrupted. It is unclear how service use, self-harm and suicide has changed since the pandemic started. To gain timely information, this systematic review focused on studies based on administrative data that compared psychiatric service use, self-harm and suicide before and during the pandemic among children and young people.

**Methods and finding:**

A systematic review of studies published in English from 1 January 2020 to 22 March 2021 was conducted, using the Web of Science, PubMed, Embase and PsycINFO databases. Increases or reductions in service use were calculated and compared using percentages. Of the 2,676 papers retrieved, 18 were eligible for the review and they provided data from 19 countries and regions. Most studies assessed changes during the early phase of the COVID-19 pandemic, from March to July 2020, and three assessed the changes until October 2020. Fifteen studies reported a total of 21 service use outcomes that were quantitively examined. More than three-quarters of the 21 outcomes (81%) fell by 5–80% (mean reduction = 27.9%, SD = 35%). Ten of the 20 outcomes for psychiatric emergency department (ED) services reduced by 5% to 80% (mean = 40.1%, SD = 34.9%) during the pandemic. Reductions in service use were also recorded for ED visits due to suicide ideation and self-harm, referrals to secondary mental health services, psychiatric inpatient unit admissions and patients receiving treatment for eating disorders. However, there were also some increases. Suicide rate and the number of ED visits due to suicide attempts have increased, and there was an increase in the number of treatment sessions in a service that provided telemedicine.

**Conclusion:**

Most of the studies showed reductions in the use of psychiatric services by children and young people during the early phase of the pandemic and this highlighted potential delays or unmet needs. Suicide rate has increased during the second wave of the pandemic. Further studies are needed to assess the pattern of service use in the later phases of the COVID-19 pandemic.

**Supplementary Information:**

The online version contains supplementary material available at 10.1186/s13034-022-00452-3.

## Background

The COVID-19 pandemic has affected the health of individuals and the ways that healthcare systems work. Mandatory lockdowns and quarantine periods, school closures and social restrictions have been effective in mitigating the spread of the virus, but these measures have probably increased psychiatric symptoms among children and young people. Emerging evidence suggests that psychiatric symptoms have worsened among children and young people across the globe during the COVID-19 pandemic, deteriorating their level of mental health [1–3]. There have also been reports of increased suicides in this age range during the second wave of the pandemic, in the third quarter of 2020 [4]. COVID-19 restrictions meant that psychiatric services were shut down, or restricted, and the general use of psychiatric services has decreased [5–7]. Systematic information about how services have been used by children and young people with mental health issues during the pandemic is imperative. This will help us to plan current services more effectively and mitigate the effects of the current and future phases of the pandemic.

A recent systematic review investigated the global impact that the COVID-19 pandemic has had on how the general population have used broader healthcare services, including visits, admissions, diagnostics and therapeutics [8]. It reported that the use of healthcare services had fallen by a third for various outcomes, mainly those related to physical health [8]. However, the review did not present psychiatric services separately and broken down by different age groups. Empirical studies of general populations have reported decreases in primary care psychiatric services [5, 6] and reduced referrals to secondary care psychiatric services [7]. A large-scale survey of psychiatrists across Europe reported a significant decrease in mental health services during the first wave of the COVID-19 pandemic in 2020 [9]. The use of psychiatric services by children and young people has constantly increased over the last three decades [10–16]. However, we are not aware of any systematic reviews that have specifically focused on registered psychiatric service use, self-harm and suicide by children and young people before and during the COVID-19 pandemic.

That is why this systematic review was both timely and needed. We focused on studies that used administrative data as it can provide large datasets in a timely manner. Administrative data can also give reliable information of the trends of psychiatric service use and rare events such as suicide deaths. This systematic review aimed to evaluate the existing literature on registered psychiatric services, self-harm and suicides for children and young people aged 0–24 years of age, before and during the COVID-19 pandemic. We also wanted to specifically focus on how the use of different services had changed.

## Methods

### Search strategy and selection criteria

This systematic review was conducted in accordance with the Preferred Reporting Items of Systematic Reviews and Meta-analyses (PRISMA) [17]. The review protocol was prospectively registered with the International Prospective Register of Systematic Reviews (PROSPERO registration number CRD42021238999). Comprehensive searches of electronic databases were carried out and these focused on potentially relevant studies that were published in English between 1 January 2020 and 22 March 2021. The databases that were searched were: Web of Science, PubMed, Embase and PsycINFO. Potential papers were also identified using hand searches and the backward snowballing technique [18], which involves looking at the reference lists of the selected papers. All titles identified for screening were exported to the Mendeley reference manager program. The search and screening processes were conducted by two reviewers (Wan Mohd Azam Wan Mohd Yunus; AY and Laura Kauhanen; LK). They independently screened the papers based on the titles and abstracts after removing any duplicates. Any disagreements were discussed with two senior researchers; an assistant professor and a professor (David Gyllenberg; DG and Andre Sourander; AS). Then the two reviewers (AY and LK) independently conducted full-text assessments based on the predefined inclusion and exclusion criteria. Both of the reviewers cross-checked the included papers and any disagreements were discussed and resolved with the two senior researchers. The full search terms are included in the Additional file 1: Appendix 1.

### Inclusion and exclusion criteria

Studies on services use, self-harm and suicide deaths of children and young people from 0–24 years of age were included. The World Health Organization (WHO) definition of young people (10–24 years) were used. Young people also fitted well with the period of ‘adolescence’ defined by Sawyer et al. [19], which was also 10–24 years. Studies that included distinct sub-samples of individuals aged 0–24 were included. We considered any studies that used clinical, register-based, hospital or health system administrative data, medical records or national records that reported psychiatric service use, self-harm and/or suicides data before and during the pandemic. The ‘before and during the pandemic’ period was defined as any period during the COVID-19 pandemic and at least one corresponding period in the years before the pandemic, as defined by the authors of the studies that were included. Only scientific peer-reviewed papers that were published in English were considered. Any psychiatric service use outcomes before and during the COVID-19 pandemic periods were included, such as referrals, visits and presentations, admissions, diagnostics or therapeutic services.

### Quality assessment

The quality of the studies were assessed using the National Institutes for Health Study Quality Assessment Tool for Observational Cohort and Cross-Sectional Studies [20]. The tool comprised 14 items that assessed the potential risk of selection bias, information bias, measurement bias, confounding bias, study power and the strength of the causality in the associations between the exposure and outcomes. Two reviewers (AY and LK) divided the studies in half and rated each study independently. They then rated the other half and compared the coding for agreement. Disagreements were resolved through discussions with a third reviewer (DG).

### Data extraction and synthesis

The following data were extracted from the included studies into an Excel spreadsheet: author, country, source of the data, age, before and during pandemic timeframes, any data before and during the pandemic timepoints, outcomes and key findings. Any descriptive data on the frequencies, means and percentages of the outcomes were extracted, where available. The percentage changes before and during the COVID-19 pandemic were calculated using relative service use changes, as used in other studies [21–24]. If the assessed outcomes were available in at least five studies, we quantified the crude means, standard deviations (SD) and ranges of the changes across the studies. Due to considerable heterogeneity in the design and outcomes of the included studies, we refrained from weighting results based on factors such as sample size. Where available, statistical non-significance was included as reported by the original papers. A narrative synthesis was used to synthesize all the other findings.

## Results

### Study selection and retrieval process

**Fig. 1:**
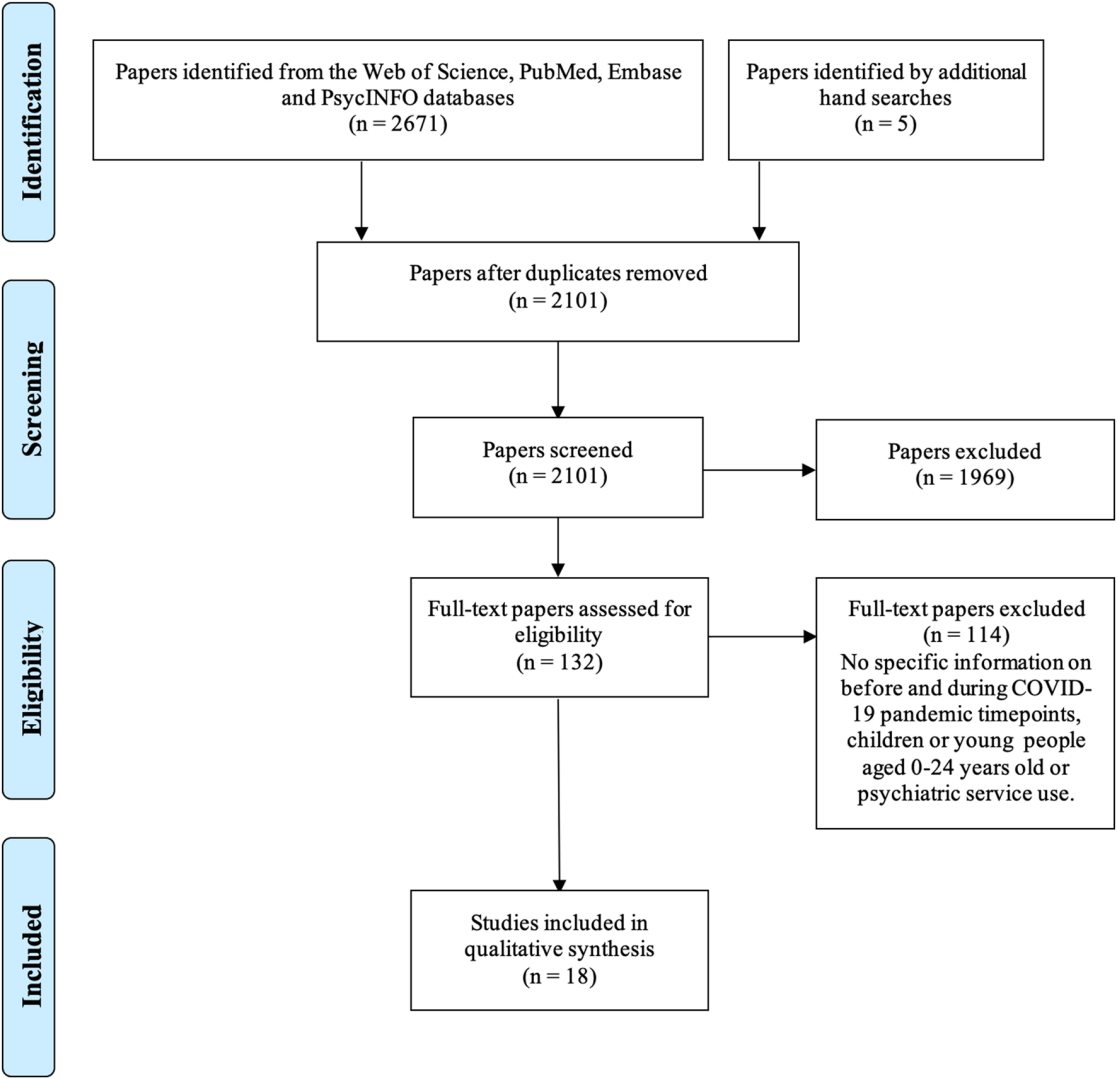
PRISMA flow diagram on how the papers were selected for the review

The initial database and manual searches identified 2676 citations and 575 duplicates were removed. The titles and abstracts of the other 2101 records were screened for eligibility, 1969 records were excluded and the full texts of 132 papers were evaluated. We excluded 114 full texts because they did not provide any timepoints to specify the periods before and during COVID-19, they did not provide any specific data or interpretation for individuals aged 0–24 or they did not focus on psychiatric service use, self-harm or suicide. At the end of this process, 18 studies were included in the qualitative synthesis. Figure 1 displays the PRISMA flow diagram for the screening and study selection processes.

### Characteristics of the included studies

#### Countries covered by the studies

Data from 19 countries and regions were included. There were 17 studies that focused on just one country and these were all conducted in developed countries: six were from the USA [22, 24–28], two from the UK [7, 29], two from Japan [4, 30], and one each from Spain [31], Italy [23], New Zealand [32], Australia [21], Canada [33], France [34] and Israel [35]. The other study comprised data from 10 different countries and regions: England, Scotland, Ireland, Austria, Italy, Hungary, Serbia, Turkey, Oman, and the United Arab Emirates [36].

#### Timepoints for measuring registered data

**Fig. 2:**
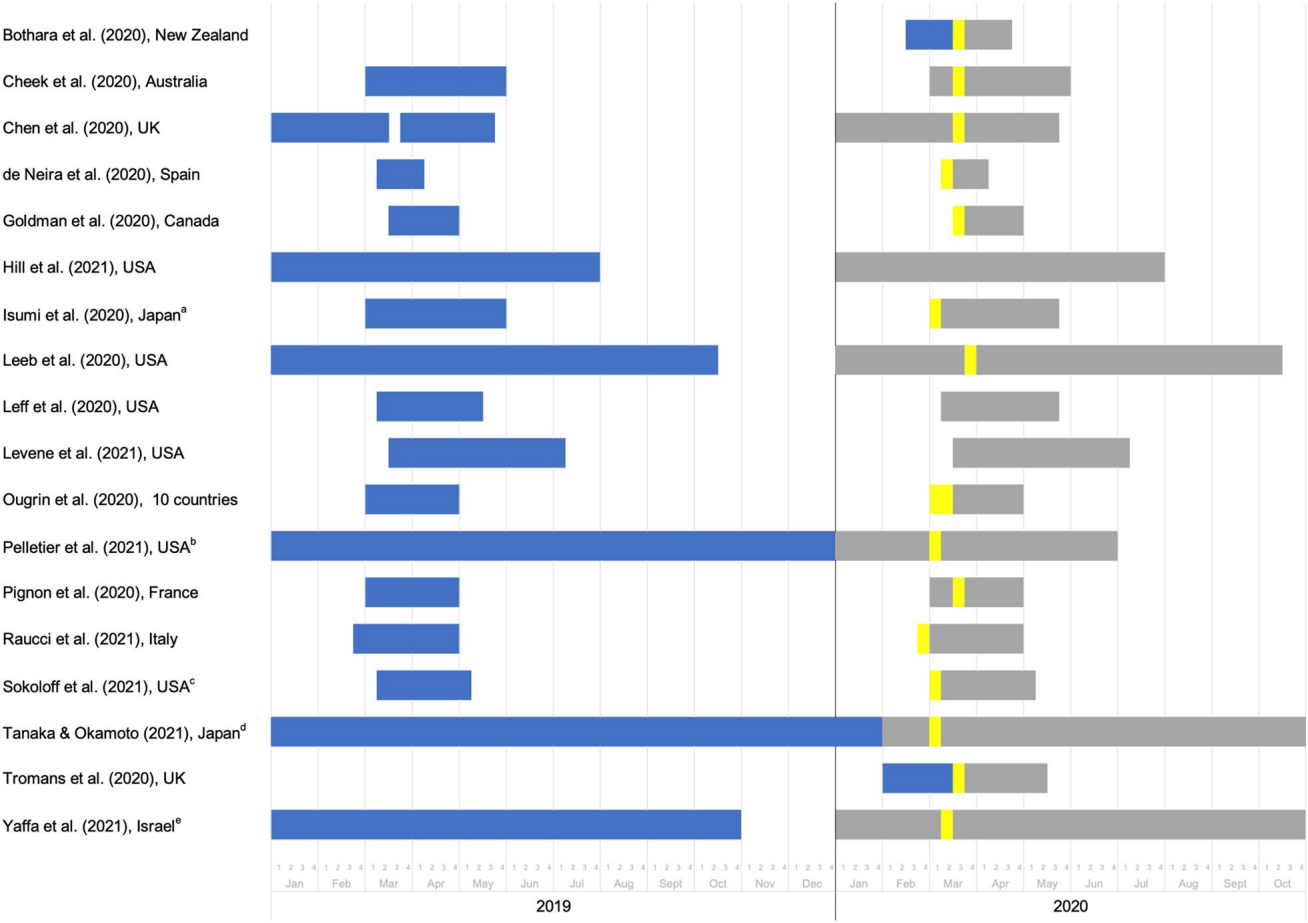
Timeframes covered by the data before (blue) and during (grey) the COVID-19 pandemic.The yellow lines depict the first lockdowns or restrictions, based on information from the study authors. ^a,b,c,d,e^Before pandemic timeframe started before January 2019: **a** March to May 2018 and 2019, **b** January to December 2010–2019, **c** 7 March to 6 May 2018 and 2019, ^d^November 2016 to January 2020, ^e^January to October 2015–2019

Figure 2 shows the time windows when psychiatric service use, self-harm or suicide data was measured before and during the COVID-19 pandemic. Most of the studies assessed the early phase of the health emergency, which was declared a pandemic by the WHO on 11 March 2020, and 15 of the studies extended the follow-up period until April to July 2020. Three studies monitored service use up to October 2020. The definition of the before and during pandemic periods varied widely between the studies. Most compared data during a COVID-19 period in 2020 and one previous year [7, 21–23, 25, 26, 28, 31, 33, 34, 36] and five used more than one previous year [4, 24, 27, 30, 35]. The other two used an earlier pre-pandemic timepoint in 2020 [29, 32]. Based on the information available in each paper, majority of the included studies defined the start of the pandemic period as the date when the earliest restrictions were imposed or each location went into lockdown. This tended to be the end of February to mid-March 2020, as depicted by the yellow lines in Fig. 2.

#### Quality assessment

It was notable that 12 of the 18 studies did not assess any potential confounding variables in their analyses. We defined these as seasonal trends or demographic differences that could be statistically measured and adjusted for in order to mitigate confounding bias. Despite this, all 18 studies were included in the review, as they were all explanatory in nature and collected data from electronic, administrative or national records. The summary of the 14 quality assessment items is in the Additional file 2: Appendix 2.

### Changes before and during the COVID-19 pandemic

**Fig. 3:**
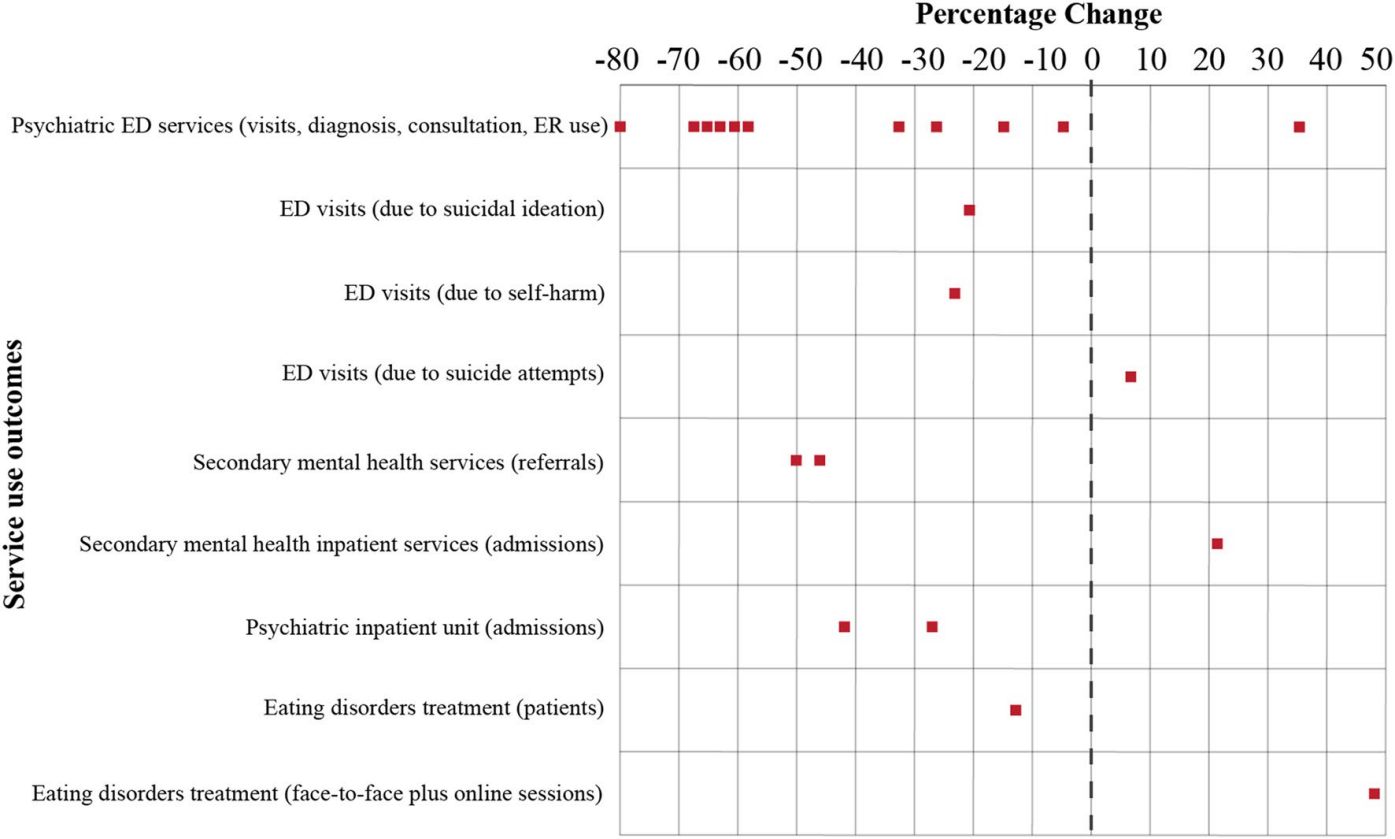
Percentage of service use changes before and during the COVID-19 pandemic. Red dots refer to percentage changes recorded by one study for one particular outcome

The key findings for each study are shown in Table 1 and Fig. 3 summarises the percentage changes for the different service use outcomes. The percentage changes are illustrated using dotted chart. The y-axis represents the service use outcomes and the x-axis is the percentage change before and during the COVID-19 pandemic. Positive percentage changes signify an increase, while negative percentage change indicate a reduction in service use, recorded by each study for that particular outcome. All except three [4, 27, 30] of the 18 studies provided descriptive data for standardized comparisons. Another study conducted at a single paediatric emergency department (ED) in New York was excluded from the quantitative analysis because it combined three different outcomes as one, namely ED visits due to suicide ideation, self-harm and suicide attempts [24]. In our review these outcomes were assessed separately. Eight studies reported more than one outcome [23–25, 29, 31, 32, 35, 36] and this meant that a total of 21 outcomes were assessed. The changes in the service use of these 21 outcomes ranged from a reduction of 80% to an increase of 48.1%, with a crude mean change of − 27.9% and a standard deviation (SD) of 35%.

**Table 1:** Summary of paper included in this review

Author, Publication date, Country	Sample/data source	Age	Numbers before pandemic	Numbers during pandemic	Percentage change	Key findings
Bothara et al. (2020), New Zealand	Paediatric patients who visited the emergency department (ED) at Christchurch Hospital	Up to 15 years of age	ED visits for psychiatric issues			Reduction in mental health diagnoses and patients presenting with suicidal thoughts, but increase in patients presenting with self-harm (sub-analysis)
			20	4	− 80%	
			ED visits due to self-harm			
			0	6	N/A	
			ED visits due to suicidal ideation			
			5	0	N/A	
Cheek et al. (2020), Australia	Paediatric patients with mental health diagnoses in four Victorian hospitals	Up to 17 years of age	ED visits for psychiatric issues			ED visits with mental health diagnoses increased (sub-analysis)
			485	656	+ 35.3%	
Chen et al. (2020), UK	General population receiving secondary care mental health clinical services from Cambridgeshire and Peterborough NHS Foundation Trust	0–19 years of age (subgroup)	Referrals to secondary mental health services (mean per day during lockdown 2020 versus same period in 2019)			Substantial reduction in the initial number of referrals per day following lockdown, but no significant acceleration after lockdown
			33.39	19	− 43.1%	
de Neira et al. (2020), Spain	Adolescents who visited the ED or were hospitalized in the Acute Inpatient Unit at the Puerta de Hierro University Hospital-Majadahonda	Up to 17 years of age Mean and SD: 14.2 ± 2.3 (pre- pandemic) and 15.36 ± 1.8 (during)	ED visits for psychiatric issues			Visits to ED and admissions to adolescent inpatient unit decreased. Average hospital stays in the adolescent inpatient unit also decreased
			64	25	− 60.9%	
			Admissions to psychiatric inpatient unit			
			31	18	− 41·9%	
			Average hospital stay (mean days)			
			14.32	8.94	− 37.6% (ns)	
Goldman et al. (2020), Canada	Pediatric patients who attended 18 EDs in British Columbia, Canada	Up to 16 years of age	Not provided	Not provided	− 41% (from original article)	ED visits for mental health issues fell by 41% (sub-analysis)
Hill et al. (2021), USA	Paediatric patients who were screened for suicide risk by a large paediatric emergency department in a major metropolitan area in Texas	11–21 years of age Mean and SD: 14.52 ± 2.22	ED visits due to suicidal ideation			Rates of suicide ideation were not uniformly higher in January to July 2020, compared to 2019. However, there were significantly higher rates of suicide ideation in March and July 2020 and higher rates of suicide attempts in February, March, April and July 2020. Rates corresponded with heightened COVID-19 restrictions. Suicide ideation was more frequent in 2020 and among females
			1134	899	− 20.7%	
			ED visits following suicide attempts			
			268	286	+ 6.7%	
Isumi et al. (2020), Japan	National suicide deaths statistics	Up to 19 years of age	Not provided	Not provided	Not provided	The first wave (March to May 2020) of the COVID-19 pandemic has not significantly affected suicide rates among children and adolescents
Leeb et al. (2020), USA	Children and adolescents attended ED based on CDC’s National Syndromic Surveillance Program (NSSP) data which include a subset of hospitals in 47 states	Up to 17 years of age	ED visits for psychiatric issues (average weekly total visits)			ED visits decreased sharply from mid-March 2020 through early April, and then increased steadily through October 2020 compared to in 2019
			3025	2872	− 5.1%	
Leff et al. (2020), USA	Paediatric patients with mental health diagnoses who presented to the paediatric ED at Yale New Haven Children’s Hospital	Up to 15 years of age	ED visits for psychiatric issues			Psychiatric-related visits were reduced. Black children were half as likely (OR = 0.55, 95% CI 0.36–0.85) to present with mental health conditions that may have signified delayed unmet needs than white children. Females continued to visit ED more than males, as they did before the pandemic period
			378	148	− 60.8%	
Levene et al. (2021), USA	Paediatric patients seen by the paediatric ED of a quarternary children’s hospital	Up to 20 years of age	ED visits for psychiatric issues			Psychiatric and psychosocial visits declined (sub-analysis)
			607	197	− 67.5%	
Ougrin et al. (2020), 10 countries and regions	Children and young people who presented with self-harm to 23 hospital EDs in 10 countries/regions: England (3 areas), Scotland (2 areas), Italy (2 areas), Ireland, Austria, Hungary, Serbia, Turkey, Oman and the United Arab Emirates	Up to 18 years of age Mean and SD 15.2 ± 2.0 (pre) and 15.4 ± 1.7 (during)	ED visits due to self-harm			ED visits for psychiatric issues were reduced, specifically emergency psychiatric presentations for self-harm. Self-harm patients were mainly female, average age 15 years old, from the countries’ dominant ethnic groups, in education, employment or training and not looked after by the local authority
			612	470	− 23.2%	
			ED visits for psychiatric issues			
			1239	834	− 32.7%	
			Admission to psychiatric inpatient units (England only)			
			753	550	− 27.0%	
Pelletier et al. (2021), USA	Patients seen by 49 paediatric hospitals in the USA	Median (Interquartile range): 5.1 years of age (0.7–13.3)	Not provided	Not provided	Not provided	Reductions in mental health admissions (sub-analysis)
Pignon et al. (2020), France	General population seen by three psychiatric emergency centres in Paris, Colombes and Créteil	16–24 years of age (subgroup)	Psychiatric ED consultations			Significant decrease in emergency psychiatric consultations
			337	120	− 64.4%	
Raucci et al. (2021), Italy	Patients seen by EDs in two children hospitals in Rome and Palidoro	Not provided	ED visits for psychiatric issues			Reduction in visits and admissions due to mental health (sub-analysis)
			216	159	− 26.3%	
			Psychiatric ED admissions			
			87	74	− 14.9% (ns)	
Sokoloff et al. (2021), USA	Patients seen by a paediatric ED in a tertiary care children’s hospital in New York City	Up to 17 years of age	Psychiatric ED visits			Reduction in visits due to mental health, but increased visits due to suicidal ideation, suicide attempts or self-harm (sub-analysis)
			497 (Mean 2018 and 2019)	180	− 63·8%	
			ED visits due to suicidal ideation, suicide attempts, or self-harm			
			11 (Mean 2018 and 2019)	22	+ 50%	
Tanaka & Okamoto (2021), Japan	National suicide deaths statistics	Up to 19 years of age (subgroup)	Not provided	Not provided	Not provided	Suicide rate among children and adolescents increased 49% in the second wave (July to October 2020) of the pandemic corresponding to after the end of the nationwide school closure
Tromans et al. (2020), UK	General population who accessed secondary mental health services run by Leicestershire Partnership NHS Trust	Child and adolescent mental health services (sub-group)	Referrals to secondary mental health services			Significantly reduced referrals to child and adolescent secondary mental health services during lockdown
			2193	1081	− 50·7%	
			Admissions to secondary mental health services			
			14	17	+ 21·4% (ns)	
Yaffa et al. (2020), Israel	Patients with eating disorders treatment seen by the Safra Children’s Hospital at the Sheba Medical Center, Tel Hashomer	6–18 years of age	Patients received eating disorder treatment			Increased in number of treatment sessions provided for eating disorders, partly due to telemedicine. Slightly fewer patients
			423 (Mean 2015–2019)	369	− 12·8%	
			Face-to-face and online sessions provided for eating disorder treatment			
			4001 (Mean 2015–2019)	5926	+ 48·1%	

*ED* emergency department, *SD* standard deviation, *ns* not significant, *N/A* not applicable

Of the 14 studies, 11 had at least one outcome related to ED services, including the number of psychiatric visits or presentations, ED use, diagnoses and consultations. Only one study recorded an increase in psychiatric visits to the ED, of 35.3% [21], while the other studies recorded reductions of between 5.1 and 80.0% [22–24, 26, 28, 31, 32, 34, 36]. The average percentage change was 40.1 ± 34.9% in psychiatric ED services during the COVID-19 pandemic when the data were compared to before the pandemic.

Two studies reported changes ED visits due to suicide ideation, self-harm and/or suicide attempts while two studies in Japan reported data on the suicide rates based on nationwide records. One study reported a reduction in total ED visits due to suicide ideation (20.7%), but a slight increase in visits due to suicide attempts (6.7%) [25]. However, the authors concluded that the rates of suicide ideation were not uniformly higher by month, from January to July 2020, compared to the same period in 2019. Despite the reduction in total ED visits due to suicide ideation in January to July 2020, compared to the same period in 2019, the rate of suicidal ideation was 1.6 times higher in March 2020 and 1.45 times higher in July 2020. This study also reported that the rates of suicide attempts were between 58 and 134% higher in February, March, April and July 2020, compared to the same months in 2019. It also reported that suicide ideation was more frequent among females in 2020 than 2019 [25]. Another study conducted in 10 different countries and regions reported a 23.2% reduction in pooled ED visits due to self-harm [36]. However, a more detailed analysis by that study showed that the number of children and young people who presented with self-harm, as a percentage of total psychiatric ED visits, increased from 50% in 2019 to 57% in 2020, with area-specific increases in 10 of the 14 areas covered by the 10 countries. Their study also found higher proportions of children and young people who had previously visited hospitals after self-harming (36% in 2020 versus 29% in 2019) or had self-harmed in the community (71% in 2020 versus 63% in 2019). One of the study conducted in Japan during the initial phase (March to May 2020) of the pandemic reported no statistically significant difference of suicide rates among children and adolescents [30]. However another recent study also in Japan with more comprehensive data reported that the suicide rate among children and adolescents has increased 49% in the second wave (July to October 2020) of the pandemic [4].

Two studies from the UK reported reductions in referrals to secondary mental health services of 43.1% [7] and 50.7% [29]. In contrast, secondary mental health inpatient services admissions had increased by 21.4%, but the increase was not statistically significant [29]. Two studies reported that the number of patients admitted to psychiatric acute inpatient units fell by 27.0% [36] and 41.9% [31]. The second study also reported a reduction in the average hospital length of stay, from 14.32 ± 10.23 to 8.94 ± 4.87 days (p = 0.08).

One study from Israel focused on a treatment centre for eating disorders. The authors reported that the number of patients reduced by 12.8%, but the number of treatment sessions increased by nearly half (48.1%) during the pandemic [33]. The authors added that the treatment centre had moved from just face-to-face sessions before the pandemic to a combination of face-to-face and online sessions during the pandemic. This explained the increased number of sessions during the pandemic.

## Discussion

Our systematic review identified 18 studies from 19 countries and regions that focused on various psychiatric service use, self-harm and suicide of children and young people during the COVID-19 pandemic. These were compared to various periods before the pandemic. The main finding was that there were reductions in the use of psychiatric services by children and young people. The most striking reductions related to ED services during the early phase of the COVID-19 pandemic. Secondly, suicide rates have increased during the second wave of the pandemic, based on the nationwide records study in Japan. Limited evidence suggested that ED visits due to suicide ideation and self-harm reduced, but visits due to suicide attempts increased among children and young people during the early COVID-19 pandemic. These findings provide essential information that can help us to plan adequate psychiatric services for children and young people during any future pandemics or crises.

Our review extends the findings of a broad systematic review on the disruptive global impact of the COVID-19 pandemic on healthcare services [8]. It demonstrates how children and young people accessed fewer psychiatric services during the COVID-19 pandemic than before the pandemic. A number of healthcare services were reduced during the early phase of the COVID-19 pandemic. Healthcare services are considered essential services, but a WHO survey carried out in summer 2020 reported that only half of the countries in its member states ensured the continuity of services for mental, neurological and substance use disorders in their national COVID-19 response programmes [37]. These approaches may have also disrupted the continuity of psychiatric services. Most of the pandemic data collected by the studies we reviewed focused on when restrictions had just been imposed in the respective countries. More research is needed to show the effects of more extended restrictions.

We consider the perceived benefits versus risks as the possible primary explanation for the findings, particularly the reduction of ED visits. In the early phase of the pandemic, authorities continuously emphasized and broadly publicized the need to stay at home, and only leave the house when necessary. This may have affected the motivation of children and young people to access psychiatric services. Parents and adolescents might perceive that the clear and quick benefit of keeping themselves, their family and their children safe at home outweighs the risks to go out and access ED services for acute psychiatric-related issues unless for severe cases. The pandemic has had a number of negative impacts on various aspects on people’s lives, including closing schools and public places, changes in work routines and how companies operate and how families have had to organize their daily lives. This forced isolation has led to feelings of helplessness, abandonment and heightened insecurity [38]. Public health measures, such as physical distancing and quarantine periods to reduce the spread of the coronavirus, may have also reduced emergency department visits and this has meant that less children and adolescents have sought help for physical and mental health conditions [39–41]. Fear of contracting the virus [38] could have played a key role in reduced healthcare visits and there are two possible pathways when it comes to children and young people. The first is negative information from teachers, peers or social media and the second is modelling, whereby individuals are influenced by the fear that their parents experience [42]. This has probably led to increased unmet needs among children and young people and delayed help-seeking, which poses capacity challenges for psychiatric services. Some psychiatric disorders can be aggravated without timely intervention and experiencing psychiatric problems during certain periods of development may predict long-term adverse outcomes [43, 44].

We also identified a study that showed how psychiatric services adapted to the pandemic. For example, one study reported that the unprecedented use of multi-professional telemedicine treatment sessions enabled them to maintain the continuity of eating disorder treatment sessions, by adding another mode of delivery to face-to-face sessions [35]. That study found that, although the average number of patients receiving treatment had fallen in 2020, compared to the average same periods from 2015 to 2019, the combined number of face-to-face plus telemedicine sessions had increased considerably (48.1%) during the COVID-19 pandemic. This ability to adapt during a public health crisis provides a glimpse of the vast potential that technological advances can offer when it comes to transforming the delivery of certain traditional psychiatric services. This increase may signpost the way to improving the cost-effectiveness of future treatment and research is needed to show how this can be sustained in the longer term. These findings support arguments that the COVID-19 pandemic has been described as an unpredictable ‘*black swan moment*’ [45]. One article stated that it will lead to a ‘*partly, though robust, shift in mental health care provision towards online prevention, treatment, and care in the near future*’ [46].

Service use changes related to suicide ideation, self-harm and/or suicide attempts were only covered by two studies [25, 36] and there were two studies on nationwide suicide deaths record in Japan [4, 30]. Several findings can be drawn from these studies. The multinational study of 10 countries and regions by Ougrin et al. reported a reduction in the actual number of ED visits due to self-harm. However, a more detailed analysis of the pooled data showed that self-harm accounted for a slightly higher proportion of psychiatric ED visits, increasing from 50% in 2019 to 57% in 2020 [36]. Hill et al. [25] reported a similar pattern. Despite the lower number of total ED visits due to suicidal ideation from January to July 2020, compared to 2019, the by-month analysis showed no uniform pattern. However, a higher rate was seen in March 2020, which corresponded to the early COVID-19 measures and the WHO declaring the outbreak a pandemic. While the total number of ED visits due to suicide attempts slightly increased, the rates were significantly higher in February, March, April and July 2020 when they were compared with the same months in 2019 [25]. It should be emphasised that these total numbers only represented the number of children and young people who went to the ED. Most young people with suicidal ideation and self-harm do not seek professional help and are more likely to seek informal help from their peers [47]. COVID-19 may have restricted social interaction with peers and reluctance to seek help from healthcare facilities, because of the fear of infection [38], may explain the reductions in ED visits. These data point to unmet needs, rather than actual reductions in needs. Previous reviews have highlighted suicidal ideation and prior attempts as major risk factors for suicide in children [48] and young people [49]. Notably, a Japanese study that was based on national statistics reported increases in suicide rates by age. It found that the rate was highest in those aged below 20 years (49%) during the second wave of the COVID-19 pandemic, compared to other age groups [4]. This could signify a worrying indicator and calls for immediate interventions to tackle suicidality in children and young people.

## Strengths and limitations

To our knowledge, this is the first systematic review to collate empirical evidence of the changes in registered psychiatric service use, self-harm and suicide deaths among children and young people aged 0–24 during the early stages of the COVID-19 pandemic, compared to before the pandemic. The review followed rigorous PRISMA guidelines. We also quantified the changes by presenting them as percentages and that allowed us to make standardized comparisons between the various service use outcomes in the selected studies across different countries. These findings are imperative at a time when children, young people and health care services continue to adapt to the ambiguities caused by the COVID-19 pandemic. This review had a number of limitations. First, only peer-reviewed papers published in English were searched for and included, which meant that potential studies in other languages and papers that had not been peer-reviewed were not included. Second, although 18 studies were included in the review, children and young people and psychiatric services were subgroups in 11 of those studies. In those cases, we only extracted the data pertaining to the use of psychiatric services by children and young people aged 0–24 years old and this did limit our interpretations of these studies. Third, 13 of the18 studies limited the before and during pandemic periods to just 2020 and 2019. Fourth, we identified limited evidence on service use outcomes beyond ED visits or presentations. In particular, only a limited number of studies assessed the following outcomes: admission to secondary mental health services, psychiatric inpatient unit admissions, diagnoses and consultations and the number of patients who received psychiatric treatment sessions and how many they required. In addition, our review only identified one study that assessed how psychotherapeutic services were used to address mental health and psychiatric issues and that was limited to just eating disorders [35]. Fifth, 17 of the 18 studies were conducted in high-income countries, based on the World Bank list. The exception was the multinational study that included data from Serbia and Turkey, which are two middle-income countries [36]. No studies included in our review that were conducted in low-income countries.

## Conclusion

This systematic review showed a considerable reduction in the use of psychiatric service use by children and young people aged 0–24 during the initial phase of the COVID-19 pandemic, compared to before the pandemic. This was despite emerging evidence that psychiatric symptoms seemed to increase among children and young people, signifying possible unmet needs or delayed access to psychiatric services. Many countries observed this pattern for different psychiatric service use outcomes. Our findings have three public health significance. First, the COVID-19 pandemic highlighted the need for active plans on reassuring children’s and young people’s access to services as the current pandemic proceeds and future pandemics and crises are possible. Second, the pandemic has challenged traditional face-to-face services, but it has also highlighted the potential of integrating technological advances into psychiatric services. Third, changes in the way that children and young people use services may also modify traditional help-seeking models. Further research is needed on how to improve the efficient use of psychiatric services among children and young people and how these services can be maintained.

## Supplementary Information

**Additional file 1**. Search terms.

**Additional file 2**. Quality assessment of the studies included in the review.

## Data Availability

Not applicable.
