# Supplementary material for: Registered psychiatric service use, self-harm and suicides of children and young people aged 0–24 before and during the COVID-19 pandemic: a systematic review
Source: Child Adolesc Psychiatry Ment Health. 2022 Feb 25;16:15. doi: 10.1186/s13034-022-00452-3 (PMC8874300; doi:10.1186/s13034-022-00452-3)
Supplement: Supplementary file 1 — Additional file 1. Search terms. [file 13034_2022_452_MOESM1_ESM.docx]

**Additional file 1 - Search terms**

**Web of Science (1 January 2020 to 22 March 2021)**

| **Themes** | **Search string** |
| --- | --- |
| **COVID** | TS=(covid* OR coronavirus* OR nCOV OR “SARS-CoV-2” OR quarant* OR lockdown OR pandemic* OR outbreak) AND |
| **Service use or mental health** | TS=(psychiatr* OR psycholog* OR neuropsych* OR mental* OR psychopatholog* OR depress* OR anxi* OR phobia* OR autis* OR ASD OR “attention deficit*” OR stress* OR “posttraumatic stress” OR PTSD OR wellbeing OR well-being OR “quality of life” OR mood* OR insomnia OR suicid* OR “self-harm” OR “self-injury” OR externali* OR internali* OR service* OR admit* OR admission*) AND |
| **Children or young person** | TS=(Infant* OR Child* OR Toddler* OR Adolescen* OR Teen* OR Paediatric* OR Pediatric* OR School* OR “young adult*” OR “young people” OR “young person” OR preteen OR youth* OR student*) AND |
| **Before and during** | TS=(“time trend” OR “time-trend” OR trend OR trends OR “before and during” OR “before and after” OR “cross-sectional” OR longitudinal OR panel OR cohort OR epidemiolog* OR compar* OR differen* OR change* OR before OR registry OR register*) |
| **Papers identified** | **1652** |

**PubMed (1 January 2020 to 22 March 2021)**

| **Themes** | **Search string** |
| --- | --- |
| **COVID** | "COVID-19"[Mesh] OR "SARS-CoV-2"[Mesh] OR covid*[tiab] OR coronavirus*[tiab] OR nCOV[tiab] OR SARS-CoV-2[tiab] OR quarant*[tiab] OR lockdown[tiab] OR pandemic*[tiab] OR outbreak[tiab] AND |
| **Service use or mental health** | "Psychiatric Department, Hospital"[Mesh] OR "Hospitals, Psychiatric"[Mesh] OR "Emergency Services, Psychiatric"[Mesh] OR "Psychiatric Rehabilitation"[Mesh] OR "Psychosocial Intervention"[Mesh] OR "Mental Health"[Mesh] OR psychiatr*[tiab] OR psycholog*[tiab] OR neuropsych*[tiab] OR mental*[tiab] OR psychopatholog*[tiab] OR depress*[tiab] OR anxi*[tiab] OR phobia*[tiab] OR autis*[tiab] OR ASD[tiab] OR “attention deficit*”[tiab] OR stress*[tiab] OR "posttraumatic stress"[tiab] OR PTSD[tiab] OR wellbeing[tiab] OR "well-being" [tiab] OR “quality of life”[tiab] OR mood*[tiab] OR insomnia[tiab] OR suicid*[tiab] OR "self-harm" [tiab] OR "self-injury" [tiab] OR externali*[tiab] OR internali*[tiab] OR service*[tiab] OR admit*[tiab] OR admission[tiab] AND |
| **Children or young person** | "Child, Preschool"[Mesh] OR "Child"[Mesh] OR "Adolescent"[Mesh] OR "Young Adult"[Mesh] OR Infant*[tiab] OR Child*[tiab] OR Toddler*[tiab] OR Adolescen*[tiab] OR Teen*[tiab] OR Paediatric*[tiab] OR Pediatric*[tiab] OR School*[tiab] OR “young adult*”[tiab] OR “young people”[tiab] OR “young person”[tiab] OR preteen[tiab] OR youth*[tiab] OR student*[tiab] AND |
| **Before and during** | "Epidemiologic Studies"[Mesh] OR “time trend”[tiab] OR "time-trend"[tiab] OR trend[tiab] OR trends[tiab] OR “before and during”[tiab] OR “before and after”[tiab] OR “cross-sectional”[tiab] OR longitudinal[tiab] OR panel[tiab] OR cohort[tiab] OR epidemiolog*[tiab] OR compar*[tiab] OR differen*[tiab] OR change*[tiab] OR before[tiab] OR registry[tiab] OR register*[tiab] |
| **Papers identified** | **333** |

**EMBASE (1 January 2020 to 22 March 2021)**

| **Themes** | **Search string** |
| --- | --- |
| **COVID** | covid*:ti,ab OR coronavirus*:ti,ab OR nCOV:ti,ab OR SARS-CoV-2:ti,ab OR quarant*:ti,ab OR lockdown:ti,ab OR pandemic*:ti,ab OR outbreak:ti,ab AND |
| **Service use or mental health** | psychiatr*:ti,ab OR psycholog*:ti,ab OR neuropsych*:ti,ab OR mental*:ti,ab OR psychopatholog*:ti,ab OR depress*:ti,ab OR anxi*:ti,ab OR phobia*:ti,ab OR autis*:ti,ab OR ASD:ti,ab OR ‘attention deficit*’:ti,ab OR stress*:ti,ab OR ‘posttraumatic stress’:ti,ab OR PTSD:ti,ab OR wellbeing:ti,ab OR ‘well-being‘:ti,ab OR ‘quality of life’:ti,ab OR mood*:ti,ab OR insomnia:ti,ab OR suicid*:ti,ab OR ‘self-harm‘:ti,ab OR ‘self-injury‘:ti,ab OR externali*:ti,ab OR internali*:ti,ab OR service*:ti,ab OR admit*:ti,ab OR admission:ti,ab AND |
| **Children or young person** | Infant*:ti,ab OR Child*:ti,ab OR Toddler*:ti,ab OR Adolescen*:ti,ab OR Teen*:ti,ab OR Paediatric*:ti,ab OR Pediatric*:ti,ab OR School*:ti,ab OR ‘young adult*’:ti,ab OR ‘young people’:ti,ab OR ‘young person’:ti,ab OR preteen:ti,ab OR youth*:ti,ab OR student*:ti,ab AND |
| **Before and during** | ‘time trend’:ti,ab OR ‘time-trend’:ti,ab OR trend:ti,ab OR trends:ti,ab OR ‘before and during’:ti,ab OR ‘before and after’:ti,ab OR ‘cross-sectional’:ti,ab OR longitudinal:ti,ab OR panel:ti,ab OR cohort:ti,ab OR epidemiolog*:ti,ab OR compar*:ti,ab OR differen*:ti,ab OR change*:ti,ab OR before:ti,ab OR registry:ti,ab OR register*:ti,ab |
| **Papers identified** | **549** |

**PsychINFO (1 January 2020 to 22 March 2021)**

| **Themes** | **Search string** |
| --- | --- |
| **COVID** | TI (covid* OR coronavirus* OR nCOV OR “SARS-CoV-2” OR quarant* OR lockdown OR pandemic* OR outbreak) OR AB (covid* OR coronavirus* OR nCOV OR “SARS-CoV-2” OR quarant* OR lockdown OR pandemic* OR outbreak) AND |
| **Service use or mental health** | TI (psychiatr* OR psycholog* OR neuropsych* OR mental* OR psychopatholog* OR depress* OR anxi* OR phobia* OR autis* OR ASD OR “attention deficit*” OR stress* OR “posttraumatic stress” OR PTSD OR wellbeing OR well-being OR “quality of life” OR mood* OR insomnia OR suicid* OR “self-harm” OR “self-injury” OR externali* OR internali* OR service* OR admit* OR admission*) OR AB (psychiatr* OR psycholog* OR neuropsych* OR mental* OR psychopatholog* OR depress* OR anxi* OR phobia* OR autis* OR ASD OR “attention deficit*” OR stress* OR “posttraumatic stress” OR PTSD OR wellbeing OR well-being OR “quality of life” OR mood* OR insomnia OR suicid* OR “self-harm” OR “self-injury” OR externali* OR internali* OR service* OR admit* OR admission*) AND |
| **Children or young person** | TI (Infant* OR Child* OR Toddler* OR Adolescen* OR Teen* OR Paediatric* OR Pediatric* OR School* OR “young adult*” OR “young people” OR “young person” OR preteen OR youth* OR student*) OR AB (Infant* OR Child* OR Toddler* OR Adolescen* OR Teen* OR Paediatric* OR Pediatric* OR School* OR “young adult*” OR “young people” OR “young person” OR preteen OR youth* OR student*) AND |
| **Before and during** | TI (“time trend” OR “time-trend” OR trend OR trends OR “before and during” OR “before and after” OR “cross-sectional” OR longitudinal OR panel OR cohort OR epidemiolog* OR compar* OR differen* OR change* OR before OR registry OR register*) OR AB (“time trend” OR “time-trend” OR trend OR trends OR “before and during” OR “before and after” OR “cross-sectional” OR longitudinal OR panel OR cohort OR epidemiolog* OR compar* OR differen* OR change* OR before OR registry OR register*) AND |
| **Papers identified** | **137** |
