# Supplementary material for: Registered psychiatric service use, self-harm and suicides of children and young people aged 0–24 before and during the COVID-19 pandemic: a systematic review
Source: Child Adolesc Psychiatry Ment Health. 2022 Feb 25;16:15. doi: 10.1186/s13034-022-00452-3 (PMC8874300; doi:10.1186/s13034-022-00452-3)
Supplement: Supplementary file 2 — Additional file 2. Quality assessment of the studies included in the review. [file 13034_2022_452_MOESM2_ESM.docx]

**Additional file 2 – Quality assessment of the studies included in the review**

| **Author** | **Q1** | **Q2** | **Q3** | **Q4** | **Q5** | **Q6** | **Q7** | **Q8** | **Q9** | **Q10** | **Q11** | **Q12** | **Q13** | **Q14** |
| --- | --- | --- | --- | --- | --- | --- | --- | --- | --- | --- | --- | --- | --- | --- |
| Bothara et al. (2020) | **Y** | **Y** | **N/A** | **Y** | **N/A** | **N/A** | **Y** | **N/A** | **Y** | **Y** | **Y** | **Y** | **N/A** | **N** |
| Cheek et al. (2020) | **Y** | **Y** | **N/A** | **Y** | **N/A** | **N/A** | **Y** | **N/A** | **Y** | **Y** | **Y** | **Y** | **N/A** | **N** |
| Chen et al. (2020) | **Y** | **Y** | **N/A** | **Y** | **N/A** | **N/A** | **Y** | **N/A** | **Y** | **Y** | **Y** | **Y** | **N/A** | **Y** |
| de Neira et al. (2020) | **Y** | **Y** | **N/A** | **Y** | **N/A** | **N/A** | **Y** | **N/A** | **Y** | **Y** | **Y** | **Y** | **N/A** | **N** |
| Goldman et al. (2020) | **Y** | **Y** | **N/A** | **Y** | **N/A** | **N/A** | **Y** | **N/A** | **Y** | **Y** | **Y** | **Y** | **N/A** | **N** |
| Hill et al. (2021) | **Y** | **Y** | **N/A** | **Y** | **N/A** | **N/A** | **Y** | **N/A** | **Y** | **Y** | **Y** | **Y** | **N/A** | **Y** |
| Isumi et al. (2020) | **Y** | **Y** | **N/A** | **Y** | **N/A** | **N/A** | **Y** | **N/A** | **Y** | **Y** | **Y** | **Y** | **N/A** | **Y** |
| Leeb et al. (2020) | **Y** | **Y** | **N/A** | **Y** | **N/A** | **N/A** | **Y** | **N/A** | **Y** | **Y** | **Y** | **Y** | **N/A** | **N** |
| Leff et al. (2020) | **Y** | **Y** | **N/A** | **Y** | **N/A** | **N/A** | **Y** | **N/A** | **Y** | **Y** | **Y** | **Y** | **N/A** | **N** |
| Levene et al. (2021) | **Y** | **Y** | **N/A** | **Y** | **N/A** | **N/A** | **Y** | **N/A** | **Y** | **Y** | **Y** | **Y** | **N/A** | **N** |
| Ougrin et al. (2020) | **Y** | **Y** | **N/A** | **Y** | **N/A** | **N/A** | **Y** | **N/A** | **Y** | **Y** | **Y** | **Y** | **N/A** | **Y** |
| Pelletier et al. (2021) | **Y** | **Y** | **N/A** | **Y** | **N/A** | **N/A** | **Y** | **N/A** | **Y** | **Y** | **Y** | **Y** | **N/A** | **Y** |
| Pignon et al. (2020) | **Y** | **Y** | **N/A** | **Y** | **N/A** | **N/A** | **Y** | **N/A** | **Y** | **Y** | **Y** | **Y** | **N/A** | **N** |
| Raucci et al. (2021) | **Y** | **Y** | **N/A** | **Y** | **N/A** | **N/A** | **Y** | **N/A** | **Y** | **Y** | **Y** | **Y** | **N/A** | **N** |
| Sokoloff et al. (2021) | **Y** | **Y** | **N/A** | **Y** | **N/A** | **N/A** | **Y** | **N/A** | **Y** | **Y** | **Y** | **Y** | **N/A** | **N** |
| Tanaka et al. (2021) | **Y** | **Y** | **N/A** | **Y** | **N/A** | **N/A** | **Y** | **N/A** | **Y** | **Y** | **Y** | **Y** | **N/A** | **Y** |
| Tromans et al. (2020 | **Y** | **Y** | **N/A** | **Y** | **N/A** | **N/A** | **Y** | **N/A** | **Y** | **Y** | **Y** | **Y** | **N/A** | **N** |
| Yaffa et al. (2021) | **Y** | **Y** | **N/A** | **Y** | **N/A** | **N/A** | **Y** | **N/A** | **Y** | **Y** | **Y** | **Y** | **N/A** | **N** |

Summary of risk of bias assessment for 18 included studies using the National Institutes for Health (NIH) Study Quality Assessment Tool for Observational Cohort and Cross-Sectional Studies items: Q1. Clearly stated research question/objective, Q2. Clearly specified/defined population, Q3. Participation rate ≥ 50%, Q4. Groups recruited from the same population and uniform eligibility criteria, Q5. Sample size justification, Q6. Exposure measured prior to the outcome, Q7. Sufficient timeframe to see an effect, Q8. Different levels of the exposure of interest, Q9. Valid and reliable measurement of exposure, Q10. Assessment of exposure

more than once, Q11. Valid and reliable measurement of outcome, Q12. Blinding of outcome assessors, Q13. Loss to follow-up ≤ 20%, Q14. Measured and statistically adjustment of confounding variables
